# Primary eye care: the WHO perspective

**Published:** 2022-03-01

**Authors:** Andreas Mueller, Stuart Keel, Alarcos Cieza

**Affiliations:** 1 Consultant: Sensory Functions, Disability and Rehabilitation Unit, World Health Organization, Geneva, Switzerland.; 2 Technical officer: Sensory Functions, Disability and Rehabilitation Unit, World Health Organization, Geneva, Switzerland.; 3 Unit Head: Sensory Functions, Disability and Rehabilitation Unit, World Health Organization, Geneva, Switzerland.

**The World Health Organization is committed to the integration of eye care at primary health level so that the unmet need in every country can be addressed in primary care facilities that can be accessed by all, rather than inside overused eye hospitals in urban areas.**

**Figure:**
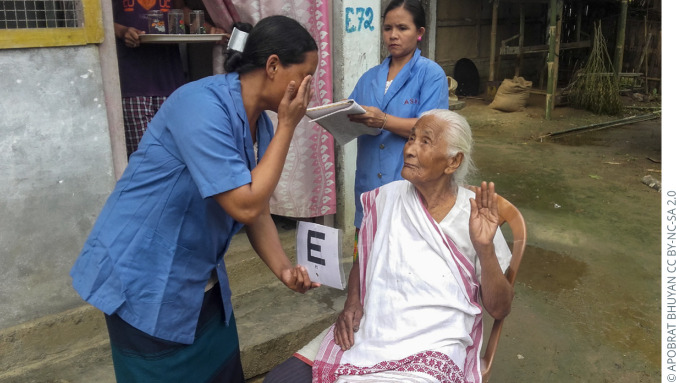
Vision assessment by government primary health care workers in Arunachal Pradesh. **INDIA**

Commitment to equity and leaving no one behind in health care is captured in Target 3.8 of the United Nations’ Sustainable Development Goal 3 (Ensure healthy lives and promote well-being for all at all ages), which is about achieving universal health coverage. Universal health coverage means that all individuals receive the health services they need without experiencing financial hardship as a result.

Although services at primary health care level have the greatest potential to reach the most underserved populations and improve equity of access to universal health coverage, it is commonly the most neglected of the levels of health care. This is also true for the delivery of eye care at primary health care level.

Recently, however, there has been a renewed focus on primary health care. In the Declaration of Astana in 2018,^1^ World Health Organization (WHO) Member States called for a renewal of the commitment to strengthen primary health care in order to deal effectively with current and future challenges to health. This focus is critical for a number of reasons:

- Primary health care enables the health system to adapt and respond to a complex and rapidly changing world with growing and ageing populations.
- With its emphasis on promotion and prevention, and a people-centred approach, primary health care has proven to be a highly effective and efficient way to address the main causes of, and risk factors for, poor health.
- Universal health coverage and the health-related Sustainable Development Goals can only be sustainably achieved with a stronger emphasis on primary health care.

## The call to integrate eye care into primary health care

The view of WHO on the importance of strengthening the integration of eye care within primary health care could not be clearer. In October 2019, WHO launched the first World Report on Vision, with a key recommendation being to reorient the model of care based on strong primary health care. In November 2020, Member States endorsed the recommendations of the World Report on Vision with the adoption of the resolution titled ‘Integrated people-centered eye care, including preventable vision impairment and blindness’ at the 73rd World Health Assembly.^2^ These high-level developments will provide important advocacy support for national efforts to strengthen primary eye care.

The key priority actions toward effective delivery of eye care at primary health care level should be:

- To prioritise the development of the eye care workforce to serve communities at the primary level of care
- To ensure effectively planned eye care referral systems from the primary level for timely treatment of eye conditions
- To raise community awareness about the availability of interventions.

## WHO support toward strengthening primary eye care at country level

In 2022, WHO will launch the Eye Care Guide for Action that will include a package of technical tools to assist governments through a phased process of situation assessment, strategic planning, implementation and monitoring and evaluation. As part of a broader package of eye care interventions,^3^ WHO will make recommendations for a set of low-cost, high-impact, evidence-based eye care interventions that can be easily, safely, and effectively delivered at primary-level health facilities in low-resource settings. The package of eye care interventions also contains the minimum essential equipment and consumables required to deliver the interventions. This primary eye care package promotes a task-shifting model and its recommendations must therefore be accompanied by appropriate training resources for the workforce at primary-level health facilities.

The commitment to primary health care is not new. Forty years ago, the Declaration of Alma-Ata recognised primary health care as a means of achieving the objective of health for all people of all nations. The renewed global focus on primary health care provides an opportunity for the eye care sector to promote the integration of eye care into primary health care in order to successfully address the unmet need at country level: not inside overused eye hospitals in urban areas, but in primary care facilities that can be accessed by all.
